# Potential Risk of Overlooking Biased Reporting of Vaccination against Novel Coronavirus Disease 2019: Lessons from Japan’s Experience with the Human Papillomavirus Vaccine

**DOI:** 10.31662/jmaj.2023-0037

**Published:** 2023-09-13

**Authors:** Yudai Kaneda, Mira Namba

**Affiliations:** 1 School of Medicine, Hokkaido University, Hokkaido, Japan; 2 School of Medicine, Keio University, Tokyo, Japan

**Keywords:** COVID-19, Vaccination, Adverse reaction, HPV vaccine, Japan

## Abstract

Vaccination is a crucial and evidence-based measure against novel coronavirus disease 2019 (COVID-19) implemented worldwide, including in Japan, where more than 67% of the population has received more than three doses of vaccination as of February 2023. Although some common adverse reactions of the vaccine, such as fever and arm pain, have been reported, serious adverse reactions resulting in death, such as myocarditis and Guillain-Barré syndrome, are rare. However, the biased reporting of the adverse reactions of the COVID-19 vaccine in the Japanese media has recently become more frequent, which is similar to the past biased reporting on the human papillomavirus (HPV) vaccine. Previously, the media’s biased reporting on the HPV vaccine caused public mistrust in vaccines, leading to a drop in vaccination rates in Japan. Thus, overlooking the sensational reporting of the media on the current COVID-19 vaccine may potentially influence people’s health behavior and awareness in the long run. Actually, experts worldwide agree that the COVID-19 vaccine is safe, and the media reports are inconsistent with the observations. Thus, it is necessary to urge the media to release accurate information to prevent tragedies caused by biased reporting. Moreover, it is essential to correct misinformation in reporting, disseminate accurate information from media outlets, such as Internet news and video-sharing sites, and promote awareness among people to support the vaccination rate against COVID-19.

Vaccination is a major countermeasure based on scientific evidence against the novel coronavirus disease 2019 (COVID-19) and has been implemented in various countries worldwide. In Japan, as of the end of February 2023, over 67% of the population has received more than three doses of vaccination against COVID-19 since its first administration on February 17, 2021 ^(1)^. Even after its classification change in the Infectious Diseases Control Law on May 8, 2023, Japanese Prime Minister Kishida has expressed his intention to continue providing free vaccinations for COVID-19, with reports in the press presenting a positive perspective in this respect. However, common adverse reactions to vaccines, such as fever, arm pain, and other symptoms, have been reported in more than half of the vaccinated individuals, regardless of the type or number of times the vaccine was administered ^(1)^. Furthermore, although rare, accounting for less than 0.001% of the number of vaccinations administered up to March 2023, there have been reported cases of severe adverse reactions such as myocarditis and Guillain-Barré syndrome following vaccination, which have resulted in death ^(1)^. In this context, there has been growing criticism of the COVID-19 vaccine in the Japanese media. In particular, three most popular Japanese weekly magazines published over 10 special issues on vaccine adverse reactions between December 2022 and February 2023. Additionally, on April 28, 2023, “The investigative television reporting on the COVID-19 vaccine adverse reactions” was elected for the 60th Galaxy Awards in the category of news reporting activities, which honors outstanding broadcasting programs in Japan. These trends indicate that reports emphasizing people’s concerns regarding vaccine adverse reactions are garnering attention across various media, including not only magazines but also television and newspapers. We feel a sense of crisis regarding this situation because there are similarities between the current biased reporting on the COVID-19 vaccine and the past biased reporting on the human papillomavirus (HPV) vaccination.

In Japan, the HPV vaccine became a routine vaccination covered by public funds for girls aged 12 to 16 in April 2013. However, several cases of suspected medical events, such as chronic pain and movement disorders, were reported after HPV vaccination, and the media broadly and sensationally reported them as adverse reactions. Indeed, Tsuda et al. revealed that articles in newspapers regarding the HPV vaccine from March 2013 contained more adverse reaction-related and authority-related keywords than those published before March 2013 and significantly fewer efficacy-related keywords ^(2)^. The sensational case reports catalyzed the formation of a negative tone of reporting independent of government recommendations and scientific evidence, leading to Japan’s Ministry of Health, Labor and Welfare (MHLW) almost immediately suspending public recommendations for HPV vaccination. As a result, the vaccination rate dropped sharply from over 70% to less than 1% and remained at that level for over 8 years. Thus, overlooking the media’s sensational reporting on the current COVID-19 vaccine adverse reactions may influence people’s health behavior and awareness in the long run.

Japan is known as one of the countries with the lowest vaccine confidence in the world. Thus, it is considered that the media covers the risks of vaccines because most Japanese people are distrustful of vaccines, and articles criticizing them are likely to be sympathetic to them. Of course, it is important to convey that such risks exist because there have been 1,754 cases of death after COVID-19 vaccination reported to the MHLW as of January 2023 ^(1)^. However, as mentioned above, this is less than 0.001% in frequency, and from the viewpoint of the availability heuristic ^(3)^, excessive reporting of such risks may accelerate people’s vaccine hesitancy. Because it has been pointed out that the vaccine’s effectiveness decreases over time, if people stop getting vaccinated, it may lead to more severe cases of COVID-19. Therefore, it is essential to accurately communicate the frequency of severe or fatal adverse reactions from vaccination rather than focusing on one of the severe cases.

Indeed, there is a consensus among experts worldwide that the COVID-19 vaccine is a safe vaccine. Xu et al. reported no increase in deaths from adverse reactions in the vaccination group, lower mortality from COVID-19, and lower mortality from other reasons ^(4)^. Moreover, because the relationship between vaccine administration and subsequent adverse reactions is often unclear, it is necessary to estimate the death risk for both vaccinated and unvaccinated individuals after adjusting for background factors when discussing the impact of vaccines. Thus, the Japanese media reports are inconsistent with these observations, and it is important to urge the media to correct their reporting and release accurate information so that tragedies caused by biased reporting, such as that we experienced with the HPV vaccine, will not happen again.

Notably, there is a time lag between the period of biased coverage of the HPV vaccine and the present, and it is unclear how much information from media such as newspapers and weekly magazines influences people’s awareness. Indeed, our research has revealed that information from Internet news and video-sharing sites has influenced changes in people’s intention to COVID-19 vaccination ^(5)^. Considering the current situation where a decrease in vaccination rates has been pointed out, correcting misinformation in reporting, disseminating accurate information from such media outlets, and promoting awareness among people will become increasingly important to support the vaccination rate against COVID-19 in the future.

## Article Information

### Conflicts of Interest

None

### Author Contributions

Conception and designing of the study: YK and MN.

Writing - original draft: YK.

Critical revision of the paper: MN.

All of the authors read the final draft and approved the submission.
